# MiR-155/GSK-3β mediates anti-inflammatory effect of Chikusetsusaponin IVa by inhibiting NF-κB signaling pathway in LPS-induced RAW264.7 cell

**DOI:** 10.1038/s41598-020-75358-1

**Published:** 2020-10-27

**Authors:** Yi Xin, Qin Yuan, Chaoqi Liu, Changcheng Zhang, Ding Yuan

**Affiliations:** 1 Affiliated Renhe Hospital of China Three Gorges University, Yichang, 443001 Hubei China; 2 College of Medical Science, China Three Gorges University, Yichang, 443002 Hubei China

**Keywords:** Drug discovery, Immunology

## Abstract

It has been demonstrated that Chikusetsusaponin IVa (CsIVa) possesses abundant biological activities. Herein, using LPS to establish acute inflammation model of mouse liver and cell line inflammation model, we investigated whether miR-155/GSK-3β regulated NF-κB signaling pathway, and CsIVa exerted anti-inflammatory effects by regulating miR-155/GSK-3β signaling pathway. Our results showed that LPS induced high expression of miR-155 and miR-155 promoted macrophage activation through GSK-3β. In addition, CsIVa inhibited inflammatory responses in LPS-induced mouse liver and RAW264.7 cells. Furthermore, we demonstrated that CsIVa improved the inflammatory response in LPS-induced RAW264.7 cells by inhibiting miR-155, increasing GSK-3β expression, and inhibiting NF-κB signaling pathway. In conclusion, our study reveals that CsIVa suppresses LPS-triggered immune response by miR-155/GSK-3β-NF-κB signaling pathway.

## Introduction

Macrophages are the main natural immune cells, which play an important role in inflammatory response. They are activated to produce a series of cytokines after being stimulated by the outside world. However, the continuous activation, migration, and infiltration of macrophages to the local tissue can lead to a variety of diseases, such as atherosclerosis, colitis, diabetes, fatty liver, and even tumors^1,2^. Inflammation itself is a self-regulated reaction process of the human body to infection, external stimulation, and injury. Moderate inflammation is a manifestation of stress state, which is beneficial to the human body, while excessive or persistent inflammatory reaction will lead to tissue damage and induce inflammatory-related diseases. Liver inflammation is widespread in liver diseases, which is the core of liver diseases. There are many factors of liver inflammation, the most common is viral hepatitis, mainly including hepatitis B and hepatitis A, in addition to autoimmune hepatitis, alcoholic hepatitis, and nonalcoholic hepatitis. It has been reported that ethanol can induce the damage of intestinal barrier function and form intestinal endotoxin, which can enter into the liver of alcoholics in large quantities, activate the downstream pathway, release related inflammatory factors in large quantities, and lead to damage of liver^3^. Kupffer cells play an important regulatory role in liver inflammation. Kupffer cells are inherent macrophages that settle in the hepatic sinuses. As the largest antigen-presenting cell group in the body, they account for more than 80% of the total number of monocyte macrophages in the body. There are many receptors on the surface, which can be activated by many ways. Inflammatory triggers such as lipopolysaccharide (LPS) activate Kupffer cells, which produce a large number of cytokines, such as tumor necrosis factor (TNF)-α, interleukin (IL)-1β and IL-6^4^. Therefore, improving macrophage inflammation may be an important way for the prevention and treatment of liver-related diseases.

MicroRNAs (miRNAs) are a class of endogenous noncoding single-stranded small RNAs usually composed of approximately 22 nucleotides, which are important epigenetic regulatory molecules. MiRNAs can promote messenger RNA(mRNA) degradation or repress mRNA translation at the post-transcriptional level by binding to the 3′-untranslated regions (3′-UTR) of target genes mRNA^5,6^. It has been found that miRNA is involved in the regulation of multiple cell differentiation and is related to many inflammatory diseases^7,8^. MiR-155,an important member of the miRNA family, is a well-known immunomodulatory miRNA that can be induced by LPS and control inflammatory processes in multiple cells and organs^9–12^. Collectively, these data suggest that miR-155 plays critical roles in regulating the inflammatory response through controlling lymphocytes and mediates the expression of related proteins.

Glycogen synthase kinase-3β(GSK-3β) is a kind of serine/threonine protein kinase, which can freely phosphorylate and regulate many kinds of signal regulatory proteins. GSK-3β is a point of convergence for numerous cell signaling pathways involved in various essential cellular functions, such as glycogen metabolism, cell cycle control, apoptosis, embryonic development, cell differentiation, cell motility, microtubule function, cell adhesion and inflammation. The effect of GSK-3β on inflammation is partly due to its ability to regulate the NF-κB signaling pathway^13,14^.Studies confirm that miR-155 is involved in cell proliferation and apoptosis by inhibiting the expression of GSK-3β^15,16^. All of these studies hint us that miR-155 may play critical roles in affecting the activation of macrophages through GSK-3β and NF-κB signaling pathways. Therefore, to find out the agents that can selectively regulate miR-155/GSK-3β-NF-κB signaling pathway will be highly significant for prevention and treatment of inflammation-related diseases.

*Panax japonicus* (*Panax japonicus*. C.A. Mey), also known as *Panax japonicus* and *Panax notoginseng*, has dual effects of ginseng and *Panax notoginseng.* It is the rhizome and fleshy root of *Panax japonicus*. C.A. Mey, which is a common Chinese herbal medicine in China. It has the functions of tonifying deficiency and strengthening, promoting blood circulation and removing blood stasis, hemostasis, and removing phlegm. There are six kinds of chemical components in *Panax japonicus*, including saponins, sugars, polyacetylenes, amino acids, volatile oil, and inorganic elements. The total saponin is the main effective component of *Panax japonicus*. It is extracted from the root tuber of *Panax japonicus* and divided into oleanol type, damane type, and okotil type. Oleanolane type saponins are the main components of total saponins of *Panax japonicus*, including *Panax japonicus* V, *Panax japonicus *IV a, *Panax japonicus* IV, *Panax japonicus* II^17,18^. In our previous studies, we have demonstrated that the total saponins of *Panax japonicus* and *Panax japonicus* V have anti-inflammatory effects through NF-κB pathway^19–22^. Our recent study also reveals that Chikusetsusaponin IVa (CsIVa) ameliorates high-fat diet-induced inflammation in adipose tissue of mice through inhibition of NLRP3 inflammasome activation and NF-κB signaling^23^.

Based on these studies, we boldly hypothesized that miR-155/GSK-3β-NF-κB signaling pathway plays a role in the protective effects of CsIVa in LPS-induced inflammation. To test our assumption, our present study was based on LPS induced acute liver inflammation in mice and RAW264.7 macrophage inflammatory model in vitro. We investigated the role of miR-155 and GSK-3β in regulating NF-κB signaling pathway during LPS-induced inflammation. Our results revealed that CsIVa could suppress the production of proinflammatory mediators, including NO, TNF-α, and IL-1β, by downregulating the expression level of miR-155, activating GSK-3β and inhibiting NF-κB signaling pathway. These findings provide insight into the mechanisms of CsIVa in the regulation of macrophage inflammation and a new potential treatment for inflammation-related diseases.

## Results

### LPS induced inflammatory response and miR-155 over expression in RAW264.7 Cells

**Figure 1:**
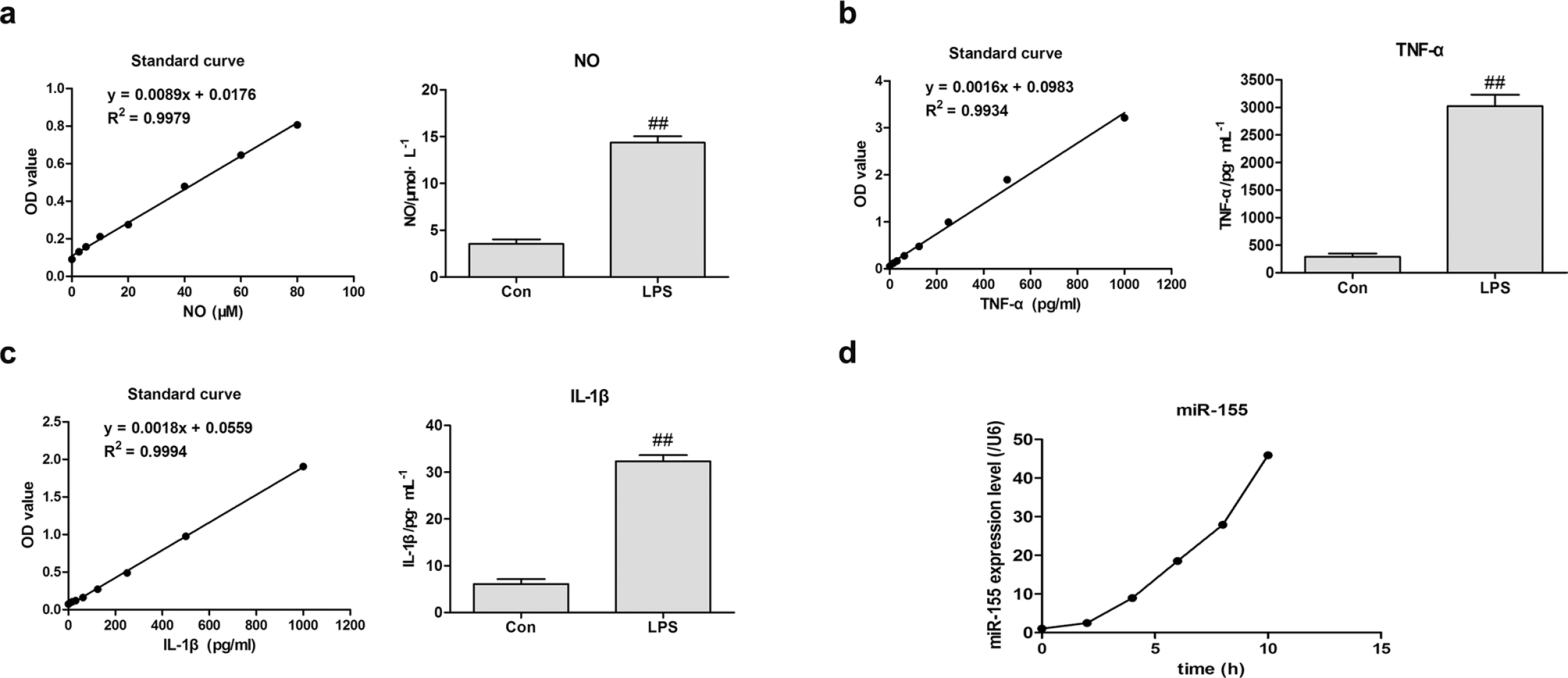
LPS treatment induces inflammatory response and miR-155 over expression in RAW264.7 Cells. (**a**) Griess reagent was used to detect the NO secretion with LPS treatment in cell supernatant. **(b,c)** ELISA kits were performed to assess respectively the levels of TNF-α **(b)** and IL-1β **(c)** with LPS treatment in cell supernatant. **(d)** Real time quantitative PCR was performed to measure the miR-155 expression with LPS treatment for the indicated time points in RAW264.7 cells. The data are expressed as the mean ± SD (*n* = 3) of results obtained from three experiments.^##^ *p* < 0.01 vs. Con group, ^*^ *p* < 0.05 and ^**^ *p* < 0.01 vs. LPS group.

LPS is a classic model of macrophage inflammatory response. To Initially explore whether we have successfully built the inflammation model, we implemented a widely used RAW264.7 macrophage inflammation model and detected the expression of NO, IL-1β, and TNF-α in RAW264.7 cells. It has been recognized that LPS increased the expression of NO, IL-1β, and TNF-α in RAW264.7 cells. Our experimental results also reached this conclusion (flammation was markedly inhibited by curcumin (Fig. 1a,b,c). We then hypothesized that miR-155 may involved in LPS-induced RAW264.7 cells and measured the effect of LPS on miR-155 expression by qRT-PCR. The results showed that higher expression levels of miR-155 in LPS-induced RAW264.7 cells was observed than in control cells (without LPS treatment) (Fig. 1d). Moreover, the expression of miR-155 significantly increased in a time-dependent manner during LPS stimulation period (Fig. 1d). This observation hints us that miR-155 might play a role in LPS-induced RAW264.7 cells.

### MiR-155/GSK-3β signaling pathway involved in the activation of LPS-induced RAW264.7 cells

**Figure 2:**
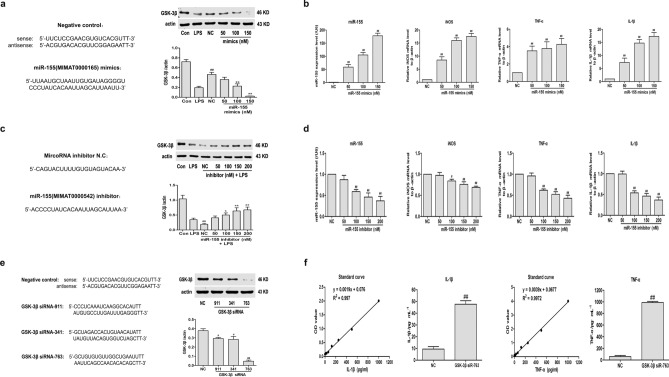
MiR-155/GSK-3β signaling pathway involved in the activation of LPS-induced RAW264.7 cells. RAW264.7 cells were transfected with miR-155 mimic, miR-155 inhibitor, GSK-3β siRNA and their correspondingly negative controls (NC) at the indicated concentrations for 48 h. **(a)** Western blot analysis was used to detect the GSK-3β expression in miR-155 mimic transfected cells and control cells. **(b)** Quantitative RT-PCR was used to measure relative expression of miR-155, iNOS, TNF-α and IL-1β in miR-155 mimic transfected cells and control cells. **(c)** Western blot analysis was performed to measure the GSK-3β expression in cells which were co-transfected with LPS and miR-155 inhibitor. **(d)** Quantitative RT-PCR was used to measure relative expression of miR-155, iNOS, TNF-α and IL-1β in cells which were co-transfected with LPS and miR-155 inhibitor. **(e)** Western blot analysis was used to detect the GSK-3β expression in cells which were transfected with GSK-3β siRNA-911, GSK-3β siRNA-341 and GSK-3β siRNA-763. **(f)** ELISA was used to measure relative expression of TNF-α and IL-1β in GSK-3β siRNA-763 transfected cells and control cells. The data are expressed as the mean ± SD (*n* = 3) of results obtained from three experiments. ^#^*p* < 0.05 and ^##^*p* < 0.01 vs. NC group.

Studies have shown that miR-155 can inhibit the expression of GSK-3β by directly binding with the 3′noncoding region of GSK-3β mRNA^15,16^. It is highly probable that GSK-3β is one downstream target gene of miR-155. To validate the GSK3β levels under the influence of miR-155, we transfected RAW264.7 cells with miR-155 mimic or inhibitor. We initially transfected various concentrations of miR-155 mimic(50, 100, 150 nM) into RAW264.7 cells to promote miR-155 expression. Our results showed that miR-155 exerted an inhibitory effect on GSK-3β activity and elevated the expression of miR-155, iNOS, TNF-α, and IL-1β in a dose-dependent manner in RAW264.7 cells, suggesting that miR-155 promotes the macrophage activation and suppress GSK-3β expression (Fig. 2a,b).

To show that LPS-induced upregulation of the above proinflammatory genes and down-regulation of GSK-3β were mediated through miR-155, the RAW264.7 cells were transfected with various concentrations of miR-155 inhibitor (50, 100, 150, 200 nM) plus LPS(1 μg/mL) to decrease miR-155 expression. Our results showed that miR-155 inhibitor reversed the downregulation of GSK-3β level and significantly decreased the expression of miR-155, iNOS, TNF-α, and IL-1β in a dose-dependent manner in LPS-induced RAW264.7 cells (Fig. 2c,d). These results suggest that inhibition of miR-155 can overregulate GSK-3β and attenuate LPS-induced inflammatory response. The above results prove that LPS activity is mediated by miR-155 and GSK-3β is also negatively regulated by miR-155 in LPS-induced RAW264.7 cells.

Subsequently, to further verify whether GSK-3β activated macrophages, we observed the expression of inflammatory factors by blocking GSK-3β. We designed three GSK-3β siRNA primers to transfect three different target sequences of GSK-3β siRNA into cells and screened out the best group of target sequences for further experiments. The results showed that GSK-3β siRNA-763 had the best inhibition effect on the expression of GSK-3β protein (Fig. 2e). Therefore, GSK-3β siRNA-763 group is selected as the best interference group. The results showed that the secretion of TNF-α and IL-1β increased significantly after GSK-3β siRNA-763 transfection compared with NC group, indicating that inhibition of GSK-3β activity can significantly promote the expression of inflammatory factors (Fig. 2f).

All in all, the above results support the hypothesis that GSK-3β is negatively regulated by miR-155 in LPS-induced RAW264.7 cells and proved indirectly that LPS activity is mediated through downregulation of GSK-3β by miR-155.

### CsIVa treatment inhibited LPS-induced inflammation in RAW264.7 cells and mice

**Figure 3:**
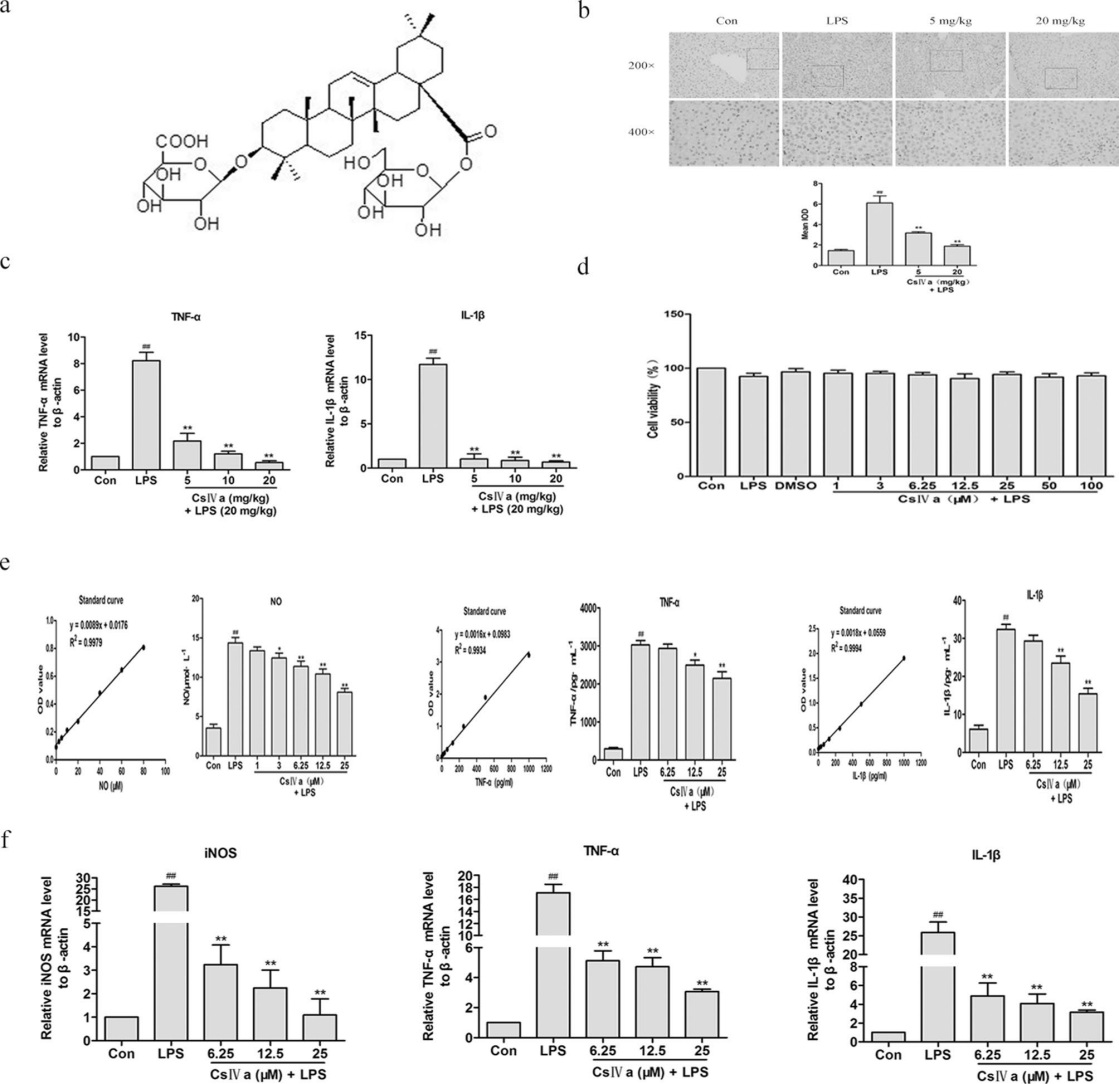
CsIVa treatment inhibited LPS-induced inflammation in RAW264.7 cells and mice. (**a)** The chemical structure of Chikusetsusaponin IVa (CsIVa); molecular weight is 794; molecular formula is C_42_H_66_O_14_. **(b)** Immunohistochemical analysis of CD68 protein expression in mice treated with LPS plus CsIVa. **(c)** The mRNA levels of TNF-α and IL-1β by Quantitative RT-PCR in liver of mice fed with LPS, LPS plus CsIVa for 4 days (*n* = 10/group). **(d)** Cell viability was examined by MTT assay and expressed relative to the DMSO control in RAW 264.7 cells. **(e)** RAW264.7 cells were pretreated with CsIVa at the indicated concentrations (6.25, 12.5, or 25 μM) for 15 h, and then stimulated with LPS (1 μg/mL) for 9 h. Levels of NO, TNF-α and IL-1β in the culture medium were assessed respectively by ELISA kits. **(f)** The mRNA expression of iNOS, TNF-α and IL-1β in LPS-stimulated RAW 264.7 cells after CsIVa pretreatment was measured by Quantitative RT-PCR. Assays were conducted three times in triplicate. Data are presented as mean ± SD (*n* = 3). ^##^*p* < 0.01 vs. Con group, *p < 0.05, and **p < 0.01 vs. LPS group.

CsIVa is the main and active component of saponins from P. japonicus (SPJ). The chemical structure of CsIVa is shown in Fig. 3a. The molecular weight of CsIVa is 794. The molecular formula of CsIVa is C_42_H_66_O_14_. To investigate whether CsIVa could exert protective effects, we carried out relevant experiments based on LPS induced acute liver inflammation in BALB/c mice in vivo and RAW264.7 macrophage inflammatory model in vitro.

In this study, according to the literature report^20^ and the previous experimental basis, we determined that the optimal time of LPS-induced liver inflammation in mice was 9 h, and the dose was 20 mg / kg. Liver function and liver index are the most basic indexes to evaluate liver injury. The serum ALT and AST levels of mice were considered to be the most commonly used indicators of liver function. As shown in Table 1, the serum ALT and AST levels and liver index in the LPS group significantly increased compared with control group. However, after CsIVa pretreatment, the serum ALT and AST levels and liver index in the CsIVa group was lower than that in the LPS group. Our results clearly showed that CsIVa alleviated LPS induced acute liver injury. The accumulation of macrophages in liver tissue plays an important role in the formation of inflammation^3^. Based on the above-described observations, we attempted to determine whether CsIVa affects the infiltration of macrophages into liver tissue and tested the effects of CsIVa on macrophage markers. CD68 is often defined as the most commonly used surface marker of Kupffer cells and is used to reflect the aggregation of macrophages in liver. We examined the effect of CsIVa on the phenotypic alteration of the Kupffer cells. PBS (pH 7.4) was used as the negative control of CD68. Brown spots indicate Kupffer cells. As shown in Fig. 3b, histological analysis showed that, compared with the Con group, the expression of CD68 protein in LPS group was significantly higher, indicating that CD68+ macrophages are actively recruited into liver tissue by LPS. Consistent with its reduction of LPS-induced increases in the serum ALT and AST levels of mice (Table 1), the significant reductions in the CD68 expression treated with CsIVa in a dose-dependent manner, as compared with LPS group (Fig. 3b). These results indicate that CsIVa treatment inhibited the accumulation of liver tissue macrophages in LPS-treated mice. In addition, the mRNA levels of TNF-α and IL-1β in the liver as determined by qRT-PCR significantly were increased after LPS induction and were decreased in a dose-dependent manner after CsIVa pretreatment (Fig. 3c), suggesting that anti-inflammatory effect of CsIVa is related to its inhibition of TNF-α and IL-1β proinflammatory cytokines. Collectively, all the above results show that CsIVa inhibited LPS-induced inflammation in mice.

**Table 1:** Effect of CsIVa pretreatment on liver index, serum ALT and AST levels in mice treated with LPS.

Groups	Control	LPS	CsIVa (mg/kg) + LPS		
			5 mg/kg	10 mg/kg	20 mg/kg
Final weight(g)	19.84 ± 1.06	22.61 ± 2.26^##^	21.05 ± 0.87	20.68 ± 0.91*	20.96 ± 0.88*
Liver quality(g)	1.09 ± 0.15	1.32 ± 0.03^##^	1.16 ± 0.04**	1.12 ± 0.07**	1.11 ± 0.07**
Liver index(%)	4.82 ± 0.21	5.87 ± 0.56^##^	5.51 ± 0.28	5.41 ± 0.41	5.28 ± 0.35**
ALT(U/L)	7.63 ± 10.86	50.42 ± 9.45^##^	36.61 ± 9.81	27.03 ± 11.31**	23.03 ± 10.83**
AST(U/L)	26.76 ± 6.51	81.23 ± 12.23^##^	45.29 ± 9.24**	36.55 ± 13.52**	29.67 ± 10.38**

The results are presented as the mean ± SD (*n* = 10).

^##^*p* < 0.01 vs control group. *p < 0.05, **p < 0.01 vs LPS group.

Then, RAW264.7 Cells were co-cultured with LPS(1 μg/ml) and CsIVa at different concentrations (1–100 μM ), and then cell viability, protein and gene levels of iNOS, TNF-α and IL-1β were detected in the present study. As shown in Fig. 3d, the LPS group had no significant effect on the cell growth compared with the con group. Moreover, our results showed that LPS stimulation significantly increased the release of NO, TNF-α, and IL-1β (Fig. 3e) and the mRNA level of iNOS, TNF-α and IL-1β (Fig. 3f) in RAW 264.7 cells, which was significantly decreased by CsIVa treatment in a dose-dependent manner. These results demonstrate that CsIVa can suppress LPS-induced inflammation in RAW264.7 Cells.

### CsIVa treatment inhibited miR-155 expression and induced GSK-3β activation and subsequent NF-κB suppression in LPS-stimulated inflammation

**Figure 4:**
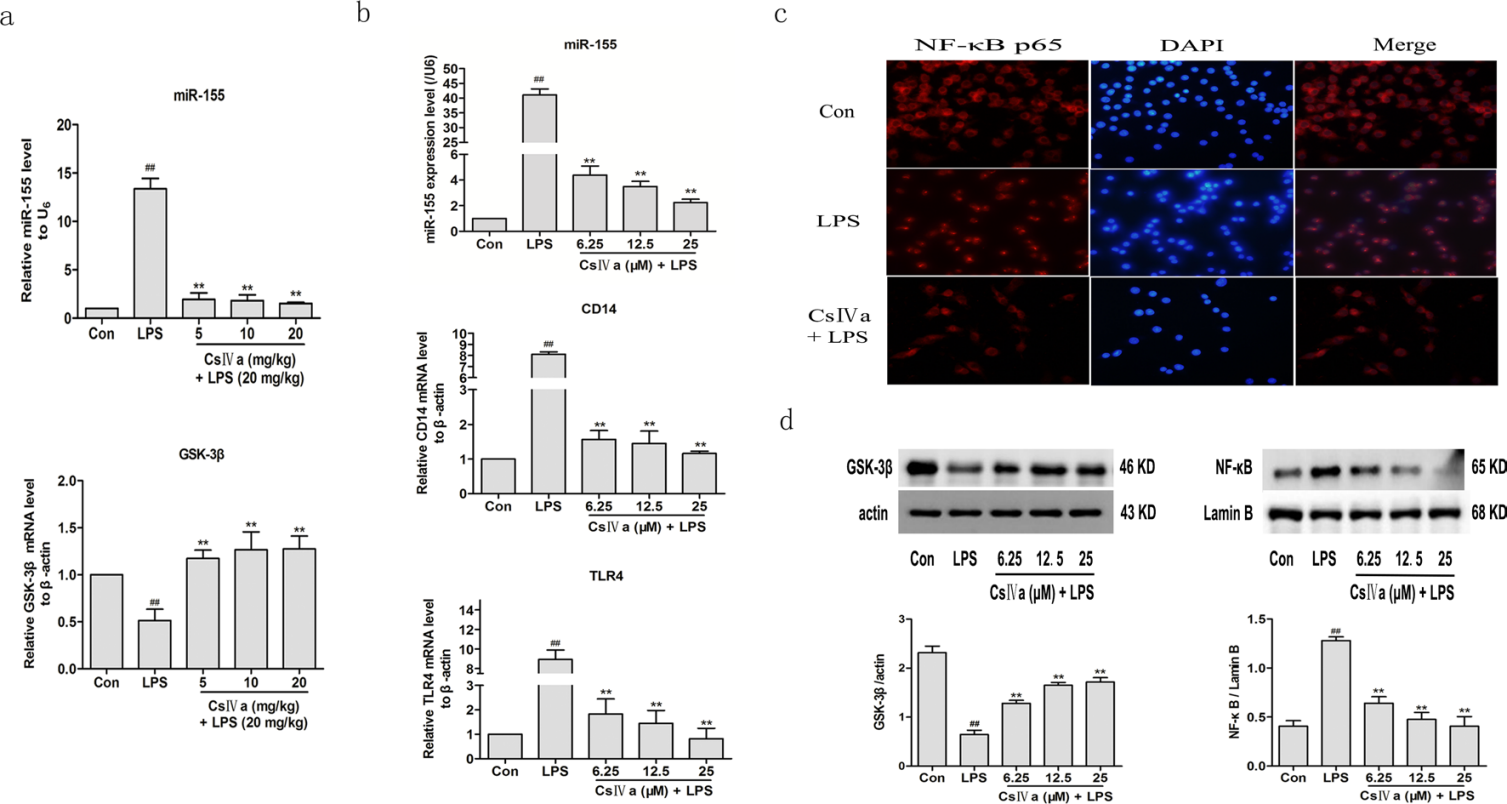
CsIVa treatment inhibited miR-155 expression and induced GSK-3β activation and subsequent NF-κB suppression in LPS-stimulated RAW 264.7 cells and mice. **(a)** Quantitative RT-PCR was used to measure relative expression of miR-155 and GSK-3β in mice which were co-treated with LPS and CsIVa. **(b)** The level of miR-155, CD14 and TLR4 were measured by quantitative RT-PCR in LPS-stimulated RAW264.7 cells. **(c)** Immunofluorescence staining of NF-κB. RAW264.7 cells were incubated with or without LPS in the presence of CsIVa. NF-κB p65 was detected by immunostaining (red); nuclear was stained by DAPI (blue). **(d)** The protein expression of GSK-3β and NF-κB were investigated using Western blot analysis in LPS-stimulated RAW264.7 cells. Three independent experiments were performed in duplicate, the data are expressed as the mean ± SD (*n* = 3). ^##^*p* < 0.01 vs. Con group, ^**^p < 0.01 vs. LPS group.

The above results show that GSK-3β is negatively regulated by miR-155 in LPS-induced RAW264.7 cells (Fig. 2) and CsIVa can suppress LPS-induced inflammation in RAW264.7 cells (Fig. 3). This observation prompted us to determine whether CsIVa treatment affects the expression of miR-155 and GSK-3β. As shown in Fig. 4a, the miR-155 mRNA level was significantly enhanced and the GSK-3β mRNA level was significantly reduced in liver tissue when induced by LPS in comparison with the Con group. However, LPS-induced enhancement of miR-155 expression were significantly suppressed and decreased GSK-3β mRNA expression level was significantly elevated when pretreated mice with CsIVa. We also measured the miR-155 expression and the protein expression level of GSK-3β in LPS-induced RAW 264.7 cells of these groups. Similar to the results of the expression levels of miR-155 and GSK-3β in mice, the protein expression level of GSK-3β was also significantly decreased and the miR-155 expression was increased in LPS group in RAW264.7 cells. Nevertheless, CsIVa decreased the increased miR-155 and increased the reduced GSK-3β in LPS-stimulated RAW264.7 cells, suggesting that CsIVa inhibited miR-155 expression and induced GSK-3β activation (Fig. 4b–d). Collectively, these data suggest that the anti-inflammatory effects of CsIVa are likely mediated by miR-155/GSK-3β signaling pathway.

The study found that the effect of GSK-3β on inflammation is partly due to its ability to regulate the NF-κB signaling pathway^13,14^. Furthermore, our previous studies demonstrate that total saponin extracts from *Panax japonicus* (SPJ), Chikusetsu saponin V, and Chikusetsu saponin IVa can inhibit NF-κB signaling^19–23^. Therefore, we further texted the effect of CsIVa on NF-κB by examining the nuclear translocation of NF-κB via Western blot analysis and immunofluorescence analysis. TLR4 is the indispensable receptor for LPS and CD14 is required for LPS recognition by TLR4. After TLR4 encounters LPS, it activates the MyD88-dependent signaling pathway, triggers the NF-κB p65 transcription response, and induces proinflammatory cytokine secretion in macrophages, dendritic cells and some epithelial cells. As shown in Fig. 4b, the expression of CD14 and TLR4 was significantly decreased in a dose-dependent manner after CsIVa treatment compared with LPS group. The Fig. 4c showed the cytoplasmic localization of NF-κB in the control cells (upper panel), the nuclear translocation of NF-κB in cells treated with LPS (middle panel) and CsIVa treatment blocked the nuclear translocation of NF-κB caused by LPS stimulation (lower panel), suggesting that the translocation of NF-κB from the cytoplasm to the nucleus in RAW24.7 cells after the LPS treatment and CsIVa decrease nuclear translocation of NF-κB induced by LPS. Additionally, we also measured the expression of NF-κB p65 in the nucleus of these groups by Western blot. Similar to the results of the NF-κB p65 expression levels in the nucleus using immunofluorescence, the NF-κB p65 expression in the nucleus was increased significantly in LPS group as compared to that in Con group, whereas CsIVa treatment significantly reduced LPS-induced increases in levels of the NF-κB p65 expression (Fig. 4d). These results indicate that CsIVa is also likely involved in inhibiting the LPS-induced activation of NF-κB signaling pathway by strongly reduced nuclear translocation of NF-κB p65.

All in all, the evidence shows that CsIVa inhibit miR-155 expression and induce GSK-3β activation and subsequent suppress NF-κB translocation in LPS-stimulated RAW264.7 cells, suggesting that CsIVa might improve the inflammatory response of RAW 264.7 cells induced by LPS through miR-155/GSK-3β-NF-κB signaling pathway.

### MiR-155/GSK-3β mediates anti-inflammatory effect of CsIVa by inhibiting NF-κB signaling pathway in LPS-induced RAW264.7 cells

**Figure 5:**
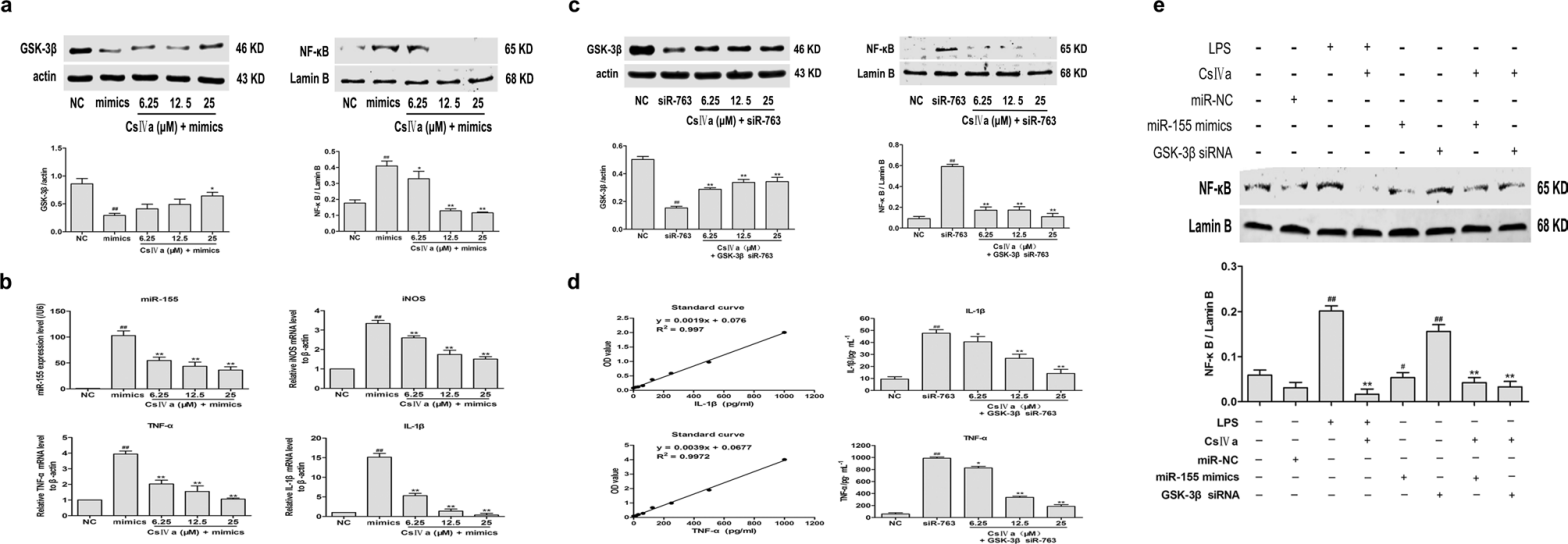
miR-155/GSK-3β mediates anti-inflammatory effect of CsIVa by inhibiting NF-κB signaling pathway in LPS-induced RAW264.7 cells. RAW264.7 cells were transfected with miR-155 mimic (100 μM) or GSK-3β siRNA-763 (100 μM) for 24 h and then treated with different concentrations of CsIVa (6.25, 12.5 and 25 μM) for additional 24 h. **(a)** Protein expression of GSK-3β and NF-κB were investigated using Western blot analysis in CsIVa-treated RAW 264.7 cells after miR-155 mimic transfection. **(b)** Quantitative RT-PCR was performed to measure the expression of miR-155, iNOS, TNF-α and IL-1β in CsIVa-treated RAW 264.7 cells after miR-155 mimic transfection. **(c)** Western blot analysis was used to detect the protein expression of GSK-3β and NF-κB in CsIVa-treated RAW 264.7 cells after GSK-3β siRNA-763 transfection. **(d)** The secretion of TNF-α and IL-1β were measured by ELISA method in CsIVa-treated RAW 264.7 cells after GSK-3β siRNA-763 transfection. **(e)** RAW264.7 cells were treated with different reagents and the nuclear protein NF-κB expression was detected. The data are expressed as the mean ± SD (*n* = 3). ^#^*p* < 0.05 and ^##^*p* < 0.01 vs. Con group or NC group, ^*^p < 0.05, ^**^p < 0.01 vs. LPS group or mimic, siRNA-763 group.

To further study the functions of CsIVa in miR-155 and GSK-3β, we transfected RAW264.7 cells with miR-155 mimics or GSK-3β siRNA. RAW264.7 cells were transfected with miR-155 mimics after CsIVa intervention and the expression of GSK-3β and NF-κB p65 by Western blot technology and the expression of miR-155, iNOS, TNF-α and IL-1β by qRT-PCR or ELISA method were measured. As shown in Fig. 5a,b, miR-155 mimics transfection alone significantly reduced the GSK-3β expression and increased the expression of NF-κB p65 in the nucleus. Meanwhile, the expression of miR-155, iNOS, TNF-α, and IL-1β was increased in miR-155 mimics transfected cells, which is consistent with the effect of LPS, suggesting that miR-155 can promote inflammation by inhibiting GSK-3β and activating NF-κB signaling pathway. After CsIVa intervention, the GSK-3β expression was increased and the expression of NF-κB p65, miR-155, iNOS, TNF-α, and IL-1β was decreased, suggesting that CsIVa could antagonize the overexpression of miR-155 and activate the inhibited GSK-3β, and inhibit NF-κB signaling pathway (Fig. 5a,b).

Moreover, the RAW264.7 cells were transfected with GSK-3β siR-763 after CsIVa intervention to detect NF-κB signaling pathway. Our results showed that the GSK-3β expression was suppressed and the expression of nuclear protein NF-κB p65 , TNF-α, and IL-1β was increased after GSK-3β siR-763 transfection alone (Fig. 5c,d). After CsIVa plus GSK-3β siR-763 treatment, the GSK-3β expression was increased and the expression of nuclear protein NF-κB p65, TNF-α and IL-1β was decreased, indicating that the decreased GSK-3β expression is increased by CsIVa and CsIVa can inhibit NF-κB signaling pathway to play an anti-inflammatory role (Fig. 5c,d). As mentioned above, CsIVa can improve inflammatory response by inhibiting overexpressed miR-155, activating silencing of GSK3β, and blocking NF-κB signaling pathway.

To further determine miR-155/GSK-3β mediates anti-inflammatory effect of CsIVa by inhibiting NF-κB signaling pathway in LPS-induced inflammation, the nuclear protein NF-κB p65 expression of RAW264.7 cells in different groups was detected by Western blot technology. As shown in Fig. 5e, LPS treatment alone significantly increased the nuclear protein NF-κB expression compared with the normal cells, and the nuclear protein NF-κB expression was significantly lower than that in the LPS alone group after CsIVa plus LPS treatment, suggesting that CsIVa can significantly block LPS activated NF-κB activity. In addition, miR-155 mimics or GSK-3β siR-763 treatment alone also increased the nuclear protein NF-κB expression compared with the miRNC group, and then the nuclear protein NF-κB expression was decreased after CsIVa intervention, suggesting that CsIVa can inhibit activated NF activity. Therefore, the above results indicate that miR-155/GSK-3β mediates anti-inflammatory effect of CsIVa by inhibiting NF-κB signaling pathway in LPS-induced RAW264.7 cells.

## Discussion

LPS can trigger inflammation in multiple organs of the body, among which the liver is the most vulnerable organ. Macrophages not only participate in the inflammatory response but also can release a variety of inflammatory mediators, such as IL-1β, TNF-α, and so on. Studies have shown that LPS can activate Kupffer cells in liver tissue after intraperitoneal injection, which release IL-1β, TNF-α, and other cytokines, and then lead to acute inflammation of liver^24^. There is a need of finding out medicines that can effectively ameliorate LPS-induced inflammation.

In the present study, we demonstrated that CsIVa could effectively ameliorate LPS-induced inflammation in mice and RAW264.7 cells. We also demonstrated an possible role of CsIVa in reducing the production of proinflammatory cytokines by suppressing miR-155 expression, enhancing the GSK-3β expression and inhibiting NF-κB transcription induced by LPS, indicating that miR-155, GSK-3β and NF-κB may be the important targets for CsIVa anti-inflammatory activity. Studies have shown that expression of miR-155 is overexpressed in macrophages induced by LPS and overexpression of miR-155 can lead to an inflammatory response^25–27^. MiR-155 is of particular interest to us because miR-155 is overexpressed in LPS-induced cells and controls inflammation responses.

In this study, we clearly demonstrated that LPS-activated RAW264.7 cells overexpressed miR-155 and LPS activity was mediated by miR-155, which is consistent with previous studies. The above studies indicate that miR-155 regulates inflammatory response and miR-155 expression is also regulated in macrophages.

Understanding the exact mechanisms by which miR-155 promotes the production of inflammatory factors may aid in determining specific therapeutic targets for improving inflammation. We all know that miR-155 is rapidly induced by LPS to promote inflammation by targeting and inhibiting various genes of the TLR4 signaling pathway, such as SOCS1^28^, SHIP1^29^ and RACK1^30^. In addition, miR-155 participates in cell apoptosis and cell proliferation through inhibiting the expression of GSK-3β by directly binding with the 3′noncoding region of GSK-3β mRNA^15,16^. Although miR-155 or GSK-3β are studied separately in inflammation^9–14^, their relationship in LPS-induced inflammation is largely unknown. Our results suggest that GSK-3β is negatively regulated by miR-155 in LPS-induced RAW264.7 cells.

Then, the mechanism by which miR-155/GSK-3β modulate the inflammatory response to LPS is needed to be further investigated. NF-κB plays a critical role in the inflammatory response and it has been traditionally used as an indicator of proinflammatory gene expression in cells exposed to bacterial infections. In the resting state, the NF-κB heterodimer (p50/p65) combined with IκB to form a trimer (p50/p65/IκB ), which existed in the cytoplasm as a potential inactive state. The presence of IκB in the trimer can inactivate NF-κB. When cells are stimulated by NF-κB activators such as TNF-α and LPS, IκB is phosphorylated by the IκB kinase (IKK) complex and dissociated from trimer, which makes the NF-κB heterodimer (p50/p65 ) exhibit NF-κB activity for nuclear translocation. The NF-κB heterodimer (p50/p65) is free to translocate to the nucleus, enhanced by DNA binding and transcriptional regulation to activate proinflammatory gene expression. In general, the activation of NF-κB is regulated at several levels, including IKK-mediated IκB degradation and nuclear translocation and DNA binding^31^. GSK-3β, as an upstream effector, was essential to inhibit the activation of NF-κB after exposure to LPS^32^. MiR-155 inhibition attenuates LPS-induced inflammation through NF-κB pathway^33–35^.

It is highly probable that GSK-3β as a negative regulator of miR-155 is involved in LPS-induced inflammation through NF-κB signaling pathway. Our results clearly confirmed that GSK-3β might be a negative regulator of miR-155. We also showed that overexpression of miR-155 or inhibition of GSK-3β activity could significantly activate NF-κB activity and increased the expression of proinflammatory cytokines. These results suggest that miR-155 may be capable of promoting inflammatory macrophage activation in vitro by targeting the negative regulator GSK-3β and activating NF-κB signaling pathway. However, it is not excluded that miR-155 activates NF-κB signaling pathway through other negative regulatory factors to mediate inflammation.

Yuan et al.^23^ demonstrate that CsIVa inhibits NF-κB pathway. Herein, we clearly demonstrated that miR-155/GSK-3β-NF-κB signaling pathway participated in LPS-induced RAW264.7 cells. Moreover, CsIVa treatment markedly inhibited miR-155 overexpression and increased GSK-3β activity in LPS-induced inflammation. Therefore, we propose that miR-155 and GSK-3β are the important targets of CsIVa for improving LPS-induced inflammation.

Many studies have found that inhibition of miR-155 attenuates inflammatory signaling in macrophages. Similarly, Our results showed that CsIVa treatment markedly suppressed the expression of miR-155 and inhibition of miR-155 could attenuate the LPS-induced expression of TNF-α, IL-1β and iNOS in RAW264.7 cells. Additionally, the inhibition of miR-155 was found to compromise the protective effects of CsIVa. Taken together, these results show, for the first time, that CsIVa exerts its inhibitory action on LPS-induced inflammation partly by suppressing miR-155 expression. Upregulation of miR-155 during inflammation is controlled in part by NF-κB^36–38^. In macrophages, apigenin reduces pr-miR-155 transcriptionally during inflammation, likely through the inhibition of NF-κB, leading to the decrease of mature miR-155^38^. We showed that CsIVa inhibits the transcriptional activity of NF-κB in mouse macrophages, suggesting that CsIVa may regulate the transcription of pri-miR-155 in an NF-κB‐mediated pathway.

Importantly, in this study, miR-155 has been shown to be capable of promoting inflammatory macrophage activation by targeting the negative regulator GSK-3β. We therefore hypothesized that GSK-3β might also be involved in the anti-inflammatory action of CsIVa. By using an miR-155 inhibitor and miR-155 mimic, miR-155 was shown to negatively regulate the expression of GSK-3β on macrophage activation. The data clearly confirm that GSK-3β may be an important target of miR-155. CsIVa is capable of upregulating GSK-3β expression in LPS-activated RAW264.7 cells. To further examine whether the inhibitory effect of CsIVa on LPS-induced inflammation is related to its regulation of GSK-3β expression, we determined the effect of GSK-3β inhibition on TNF-α and IL-1β expression. As expected, CsIVa activated silencing of GSK-3β and greatly attenuated the promoting effect of suppressed GSK-3β on TNF-α and IL-1β expression in LPS-activated RAW264.7 cells, suggesting that miR-155/GSK-3β mediates the anti-inflammatory activity of CsIVa. The mechanisms that CsIVa regulates the expression of miR-155 and GSK-3β are not yet fully understood. Further investigations are needed to determine miR-155 and GSK-3β are affected by CsIVa.

It is well known that the NF-κB signaling pathway is key to account for the expression of proinflammatory cytokines induced by LPS. The mechanism of LPS-induced inflammation is due to LPS activation promoting the interaction of TLR4 and MYD88, resulting in activation of NF-κB and production of proinflammatory factors^11^. Our results showed that CsIVa had a protective effect against LPS-induced inflammatory response by inhibiting the activation of NF-κB, which is consistent with previous research^23^.

In addition, we suspected that activated GSK-3β may have an anti-inflammatory effect because we and others have demonstrated that the activation of GSK-3β inhibited the activation of NF-κB^32,39^. However, studies have also shown that inhibition of GSK-3β can inhibit activation of NF-κB and reduce the production of downstream inflammatory factors^40–42^. We propose that there are two reasons for this phenomenon, one is due to the role of GSK-3β in the Wnt signaling cascade where it phosphorylates β-catenin resulting in its ubiquitin-mediated degradation, thus preventing its nuclear translocation^43^. The other is due to the inactivation of GSK-3β by phosphorylation, resulting in the activation of NF-κB^13^. In our study, whether the negative role of GSK-3β on NF-κB activity in RAW264.7 cells is mediated by direct phosphorylation of NF-κB components^44–46^, or by inhibiting IκB kinase (IKK) and preventing the normal phosphorylation and degradation of IκB^47,48^, or through inhibitory binding by increased nuclear β-catenin^49,50^ remains a matter of further study.

GSK-3β activity is crucial to regulate the inflammatory response, by either by promoting or inhibiting the process through the expression of proinflammatory or anti-inflammatory cytokines, which may be related to the experimental model, experimental object, experimental conditions and degree injury .Our results showed that inhibition of GSK-3β promoted the activation of NF-κB and increased the production of IL-1β and TNF-α. At the same time,our results also showed that CsIVa can activate suppressed GSK-3β, which might indicate that CsIVa could regulated the activity of NF-κB through GSK-3β to produce an anti-inflammatory effect. However, it is not excluded that CsIVa can activate NF-κB signaling pathway in other ways. GSK-3β is activated by phosphorylation of Tyrosine 216 and is inactivated by phosphorylation of Serine 9.

**Figure 6:**
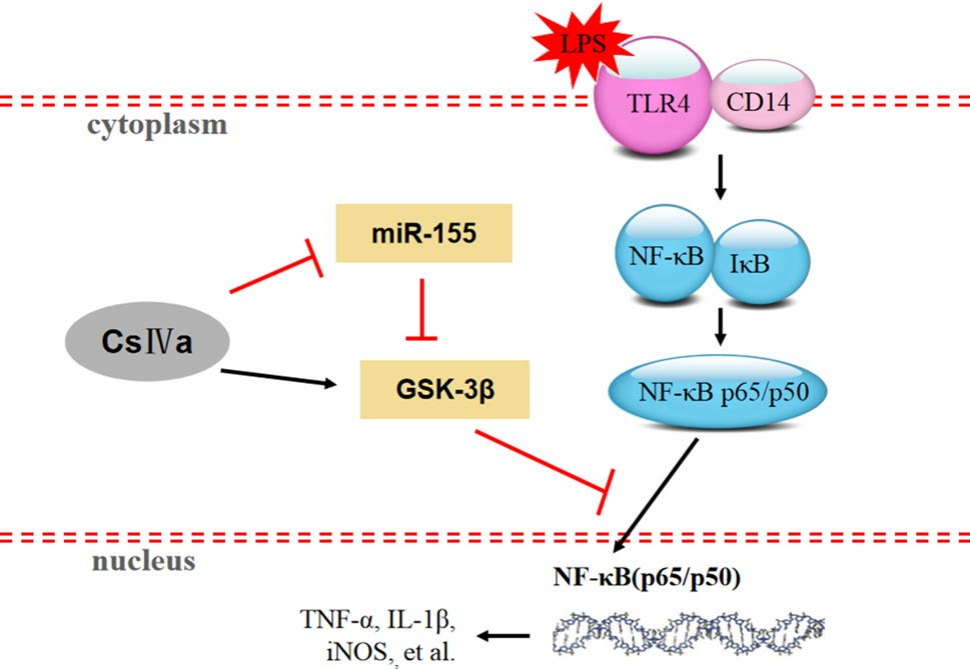
Schematic illustration of CsIVa-mediated inhibition of miR-155/GSK-3β-NF-κB signaling pathway.

Activation of macrophages is one of the important components of liver inflammation that contributes to the development of liver diseases. Activated macrophages secrete various proinflammatory mediators, chemokines, and other factors, which are believed to induce acute liver injury^51,52^. Thus, controlling their activation may ameliorate immune-mediated system disorders. Our study indicates that CsIVa can reduce inflammatory responses in LPS-activated macrophages and thus may have a potential role against liver inflammation. Furthermore, the anti-inflammatory effect of CsIVa may be due partly to the downregulation of miR-155, thus increasing the protein expression of GSK-3β and subsequently inhibiting NF-κB signaling pathway(Fig. 6).

## Conclusion

In conclusion, the present study demonstrated whether it is LPS induced acute inflammation model of mouse liver (in vivo ) or LPS induced inflammation model of RAW264.7 monocyte macrophage (in vitro), CsIVa has a good anti-inflammatory effect and can significantly inhibit the expression of TNF-α and IL-1β in LPS induced RAW264.7 macrophage and mouse liver, mainly through miR-155/GSK-3β-NF-κB signaling pathway. The results in the present study provided evidence that targeting miR-155 might be a potential therapeutic target for liver inflammation. Further animal and clinical studies are required, which may largely strengthen these findings.

## Materials and methods

### Reagents

CsIVa (purity > 98%) was purchased from Chengdu Pufei De Biotech Co., Ltd. (Chengdu, Sichuan, China). Lipopolysaccharide (LPS)(Escherichia coli 055:B5) was obtained from Sigma-Aldrich (St. Louis, MO, USA). Dulbecco’s modified Eagle’s medium (DMEM) was purchased from Gibco (Grand Island, NY, USA). Fetal bovine serum (FBS) was purchased from ScienCell company. NF-κB p65, GSK-3β, and lamin B antibodies were purchased from Cell Signaling Technology (Beverly, MA, USA). ALT and AST kits were purchased from Zhongsheng Beikong Biotechnology Co., Ltd. (Beijing, China). CD68 (GB11067) antibody was purchased from Wuhan Google Biotechnology Co., Ltd. (Wuhan, Hubei, China). β-actin and FITC labeled Goat anti mouse were purchased from Santa Cruz Biotechnology (Santa Cruz, CA, USA). NO detection kits were supplied by Beyotime Institute of Biotechnology (Jiangsu, China). Mouse tumor necrosis factor (TNF)-α and interleukin (IL)-1β enzyme-linked immunosorbent assay (ELISA) kits were purchased from Neobioscience Technology Co., Ltd. (Shenzhen, China). MiR-155 mimics, miR-155 inhibitor, GSK-3β targeted siRNA (dsRNA oligonucleotides) from GenePharma (Shanghai, China). Other cell culture reagents were purchased from Sigma-Aldrich (St. Louis, MO, USA).

### Animals and treatments

Male BALB/c mice (5–6 weeks old, 18–22 g) were provided and fed by the experimental animal center of Three Gorges University (Yichang, China) with quality certificated number SYXK 2011-0061. The experimental animals were raised in cages of 5 each. The suitable conditions of the laboratory are: temperature is 22 °C ± 2 °C, humidity is about 60. The animal protocols were approved by the Ethics Committee Guide of China Three Gorges University (Approval No: 42010200000620). All animal experiments were carried out in accordance with the Guide for the Care and Use of Laboratory Animals of China Three Gorges University.

The mice were randomly divided into five groups with 10 mice per group, fed and treated as follows: Con groups, LPS(20 mg/kg) groups, CsIVa low/medium/high dose groups (5/10/20 mg/kg) + LPS. CsIVa low/medium/high dose groups (5/10/20 mg/kg) were given by oral gavage. In the control group, the mice were given the equivalent volume of 0.9% physiological saline as used for other groups by oral gavage. In CsIVa + LPS group, the mice were orally pretreated CsIVa(5/10/20 mg/kg) in 0.9% physiological saline solution for four consecutive days. During this period, Body weight in mice was monitored and recorded daily while body weight was monitored weekly. After the last administration of CsIVa, mice in LPS group and CsIVa + LPS group were intraperitoneally injected with a single dose of LPS at 20 mg/kg, whereas the control group was treated with an equal amount of saline. Nine hours after LPS injection, all experimental animals were weighed and sacrificed, and their serum and liver samples were collected. Liver index was calculated according to the following formula: liver index = (liver weight/body weight). After weighing the liver, the largest leaf of the liver was fixed in 4% paraformaldehyde for immunohistochemistry, and the others were frozen in liquid nitrogen and stored at − 80 °C.

### Cell culture and transient transfection

Mouse RAW264.7 cells were obtained from the American Type Culture Collection (ATCC, Shanghai, China). The cells were frozen and cultured as described in previous studies^22^. For cell transfection, 1 × 10^5^ cells were seeded into petri dishes and incubated overnight and then transfected with miR-155 mimic, miR-155 inhibitor, GSK-3β siRNA and their correspondingly negative controls (NC) using Entranster-R4000 transfection reagent according to the manufacturer’s instructions. During CsIVa treatment and LPS stimulation, cells were cultured in serum-free DMEM medium.

### Serum ALT and AST activity determination

Serum alanine transaminase (ALT) and aspartate aminotransferase (AST) levels were tested using commercially available kits according to the manufacturer’s protocol.

### Immunohistochemical analysis

Paraffin-embedded mice liver tissues from all above-treated groups were cut into 5-lm thick sections. The sections were blocked with 5% BSA for 30 min and incubated with CD68 in PBS overnight at 4 °C. After washing 3 times with PBS, the sections were incubated with a biotinconjugated horseradish peroxidase secondary antibody for 50 min at room temperature. Subsequently, 3, 3-diaminobenzidene and haematoxylin were applied, respectively. Sections were examined at least five random fields, and CD68-positive foci evaluation was assessed using a Photo and Image Autoanalysis System.

### Cell viability assay

Cell viability was determined colorimetrically by MTT assay. Briefly, RAW264.7 cells were seeded in 96-well plates at a density of 3 × 10^3^ cells per well for 12 h , and pretreated with CsIVa (1, 3, 6.25, 12.5, 25, 50, 100 μM) for 1 h, then stimulated with LPS (1 μg/mL) for 24 h. After absorbing the supernatant, the 100 μL medium containing MTT (the final concentration is 0.5 g·L^-1^) was added into each well and incubated at 37 °C for 4 h. The supernatants were removed gently and the formazan crystals in each well were dissolved in 150 μL of dimethyl sulfoxide (DMSO) for 10 min at 37 °C. Then, the absorbance of each sample was read at 570 nm on a microplate reader (Bio-Rad Laboratories, Inc., Hercules, CA, USA).

### Measurement for NO production

RAW264.7 cells were seeded into 96-well plates at a density of 3 × 10^3^ cells/well for 12 h. After different treatments (LPS or CsIVa) for 24 h, NO level in the culture supernatant was collected and detected using Griess reagent following the manufacturer’s instructions. The absorbance was measured at 540 nm and nitrite concentration was calculated by comparison with a sodium nitrite solution standard curve.

### TNF-α and IL-1β assays

RAW264.7 cells were seeded into 96-well plates at a density of 3 × 10^3^ cells/well for 12 h and were treated with LPS and CsIVa or 24 h. Then the levels of TNF-α and IL-1β in the cell culture supernatants were detected using ELISA kits.

### Quantitative real-time polymerase chain reaction (qRT-PCR)

Total RNA was extracted with Trizol reagent (Invitrogen, Camarillo, CA, USA) following the manufacturer’s instructions. The reverse transcription reaction was operated with the instruction of PrimeScript RT reagent Kit of Takara company. qRT-PCR was performed using SYBR Green Master Mix kit (Takara). The amplification program was performed as follows: pre-inc-uba-tion at 95 °C, 20 s; denaturation at 95 °C, 3 s; annealing at 60 °C, 30 s; 95 °C, 15 s; and 60 °C, 60 s; followed subsequently by 40 cycle. The relative expression level of miRNAs was calculated by 2^−ΔΔCt^ method, using GAPDH or small nuclear RNA U6 as the endogenous control. The PCR primers were listed in supplementary materials (Table 2).

**Table 2:** Primer sequences of genes used in this study.

Gene	Primer sequences
miR-155	F-5′-GTCGTATCCAGTGCAGGGTCCGAGGTAT TCGCACTGGATACGACACCCCTATCAC-3′ R-5′-CGCCGCGTTAATGCTAATT-3′
U6	F-5′-CTCGCTTCGGCAGCACA-3′ R-5′-AACGCTTCACGAATTTGCGT-3′
GSK-3β	F-5′-CGGGACCCAAATGTCAAACTAC-3′ R-5′-GGAGGGATAAGGATGGTGGC-3′
iNOS	F-5′-GCCCTGCTTTGTGCGAAG-3′ R-5′-GCCCTTTGTGCTGGGAGTC-3′
β-actin	F-5′-TGCTGTCCCTGTATGCCTCT-3′ R-5′-TTTGATGTCACGCACGATTT-3′
IL-1β	F-5′-TGTCCTGTGTAATGAAAGACGGC-3′ R-5′-GCTTGTGCTGCTTGTGAGG-3′
TLR4	F-5′-CTCTGGCATCATCTTCATTGTCC-3′ R-5′-CTGCTGTTTGCTCAGGATTCG-3′
TNF-α	F-5′-TCAACCTCCTCTCTGCCGTC-3′ R-5′-GAGCAATGACTCCAAAGTAGACCTG-3′
CD14	F-5′-CAACAGGCTGGATAGGAACCC-3′ R-5′-GCCACTGCTTGGGATGATG-3′
miRNA	5′-CAGTGCAGGGTCCGAGGT-3′

### Western blot analysis

Protein extraction from the treated cells and its concentration measurement were performed as described in previous studies^22^. Denatured protein samples (30 μg/lane) were fractionated in 4 to 15% sodium dodecyl sulfate polyacrylamide gel electrophoresis (SDS-PAGE) gel and transferred to polyvinylidene difluoride (PVDF) membrane. The membrane was blocked with 5% skim milk at room temperature for 1 h, then incubated with the indicated primary antibodies specific to the target proteins overnight, and finally incubated with one of the corresponding specific secondary antibodies for 1 h. Antibodies for β-actin and Lamin B were served as the control. The membranes were exposed by chemiluminescence developing agents or scanned directly by the Odyssey CLx instrument, protein levels in each sample were evaluated by Image 6.0 software.

### Immunofluorescence analysis

RAW264.7 cells (5 × 10^5^ cells/well) were seeded into 6-well plates that contained glass coverslips. The control groups and LPS groups were pretreated with DMEM, and the CsIVa groups were pretreated with 12.5 μM CsIVa for 15 h. Then, 1 µg/ml LPS was added for another 9 h. The glass coverslips were fixed in 4% paraformaldehyde for 20 min and blocked with 5% BSA for 1 h, and then incubated with p65 NF-κB antibody overnight at 4 °C. Next, samples were incubated with fluorescent secondary antibodies (Santa Cruz, USA) in darkness at 37 °C for 1 h and counterstained with DAPI, and then mounted onto slides with glycerin. The slides were observed under a fluorescence microscope (Olympus, Tokyo, Japan).

### Statistical analysis

The data obtained from three independently repeated experiments were presented as mean ± SD. All statistical analyses were performed with GraphPad Prism 5.0. One-way analysis of variance (ANOVA) followed by least significant difference(LSD) post hoc test using SPSS18.0 software was used for comparisons between multiple groups. *p* < 0.05 was accepted as statistically significant.

## Supplementary information

Supplementary Information

## Data Availability

The data used to support the findings of this study are available from the corresponding author upon request.
