# Supplementary material for: MiR-155/GSK-3β mediates anti-inflammatory effect of Chikusetsusaponin IVa by inhibiting NF-κB signaling pathway in LPS-induced RAW264.7 cell
Source: Sci Rep. 2020 Oct 27;10:18303. doi: 10.1038/s41598-020-75358-1 (PMC7591521; doi:10.1038/s41598-020-75358-1)
Supplement: Supplementary file 1 — Supplementary Information [file 41598_2020_75358_MOESM1_ESM.pdf]

MiR-155/GSK-3β mediates anti-inflammatory effect of Chikusetsusaponin Ⅳa by inhibiting NF-κB signaling pathway in LPS-induced RAW264.7 cell

Yi Xin[1]， Qin Yuan[1*]， Chaoqi Liu[2]， Changcheng Zhang[2] & Ding Yuan[2*]

[1]Affiliated Renhe Hospital of China Three Gorges University, Yichang, Hubei, 443001, China

[2]College of Medical Science, China Three Gorges University, Yichang, Hubei, 443002, China

*Authors to whom correspondence should be addressed: Qin Yuan, E-mail: 780210109@qq.com; Ding Yuan, E-mail: yxyydyd@126.com

1. Figure 2

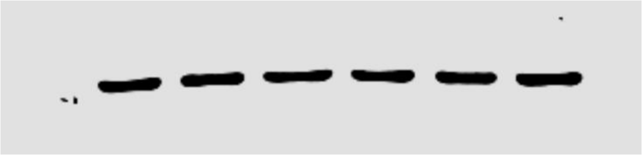

Fig. 2a actin

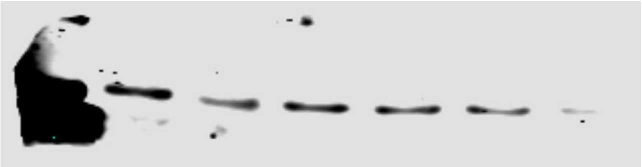

Fig. 2a GSK-3β

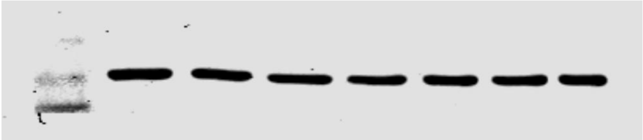

Fig. 2c actin

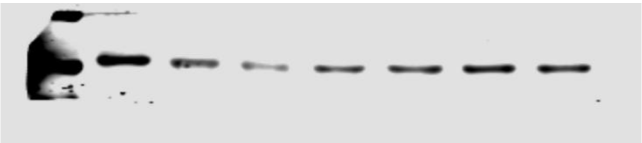

Fig. 2c GSK-3β

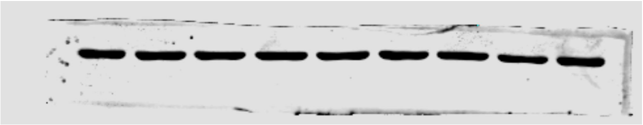

Fig. 2e actin

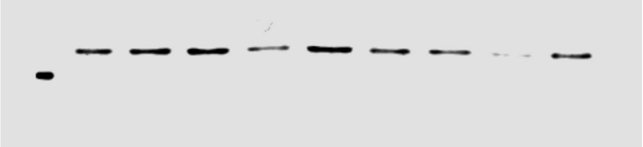

Fig. 2e GSK-3β

Figure 2e is cropped, and the original figure of figure 2e is the result of two experiments. The sequence of membrane sampling is (from left to right) : NC, GSK-3β siR-911, GSK-3β siR-341, GSK-3β siR-763, NC, GSK-3β siR-911, GSK-3β siR-341, GSK-3β siR-763, NC.

2. Figure 4

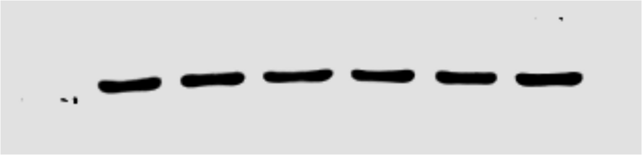

Fig. 4d actin

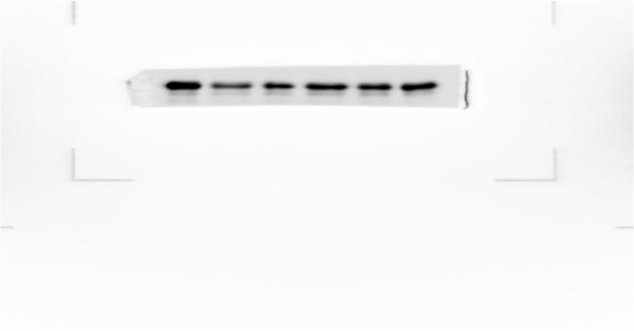

Fig. 4d GSK-3β

As is shown in Fig. 4d, the membrane of actin is scanned directly by the Odyssey CLx instrument, and the membrane of GSK-3β is exposed by chemiluminescence developing agents. The sequence of membrane sampling is (from left to right) : Con, LPS, 6.25μM, 12.5μM, 25μM, 50 μM.

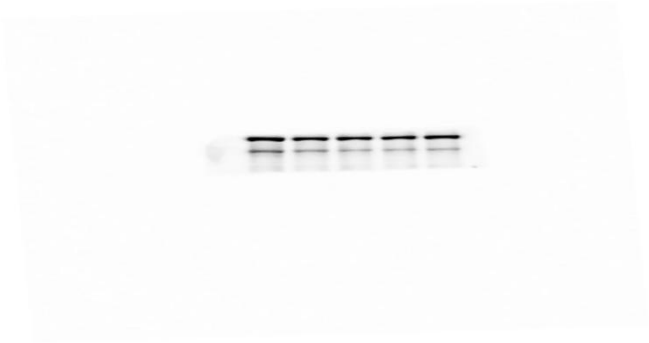

Fig. 4d LaminB

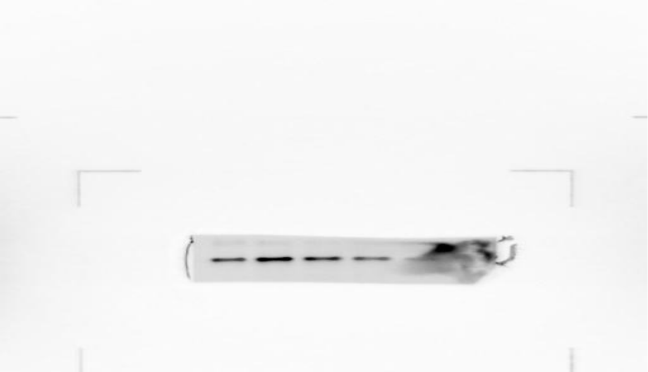

Fig. 4d NF-κB

3. Figure 5

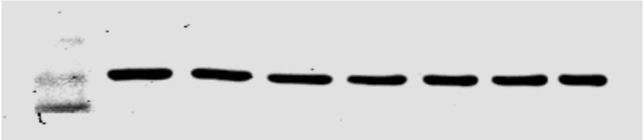

Fig. 5a actin

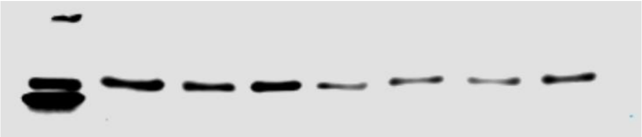

Fig. 5a GSK-3β

The sequence of membrane sampling is (from left to right) : Con, LPS, NC, mimics, 6.25 μM, 12.5 μM, 25 μM.

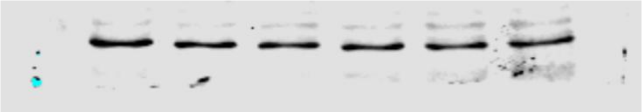

Fig. 5a LaminB

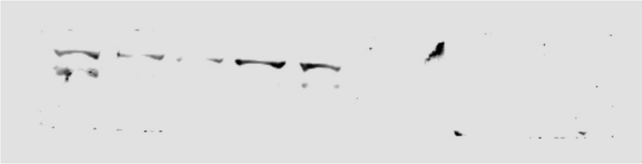

Fig. 5a NF-κB

The sequence of membrane sampling is (from left to right) : Con, NC, mimics, 6.25 μM, 12.5 μM, 25 μM.

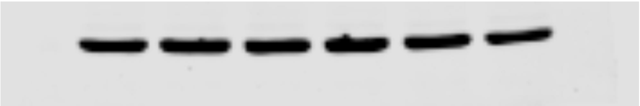

Fig. 5c actin

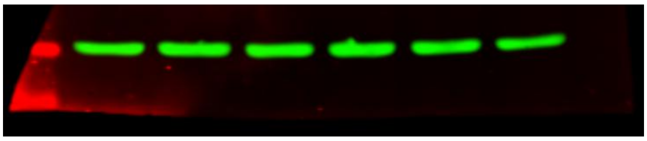

Fig. 5c actin

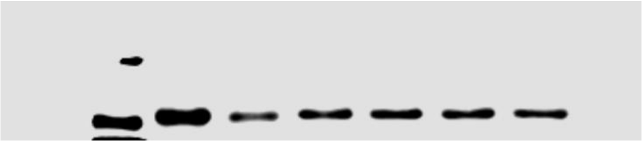

Fig. 5c GSK-3β

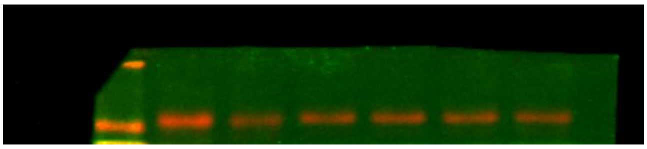

Fig. 5c GSK-3β

The sequence of membrane sampling is (from left to right) : NC, siR-763, 6.25 μM, 12.5 μM, 25 μM, 50 μM.

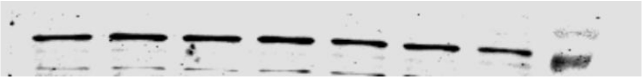

Fig. 5c LaminB

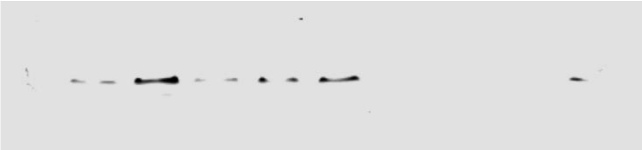

Fig. 5c NF-κB

The sequence of membrane sampling is (from left to right) : NC, siR-763, 6.25 μM, 12.5 μM, 25 μM, 50 μM, 25 μM.

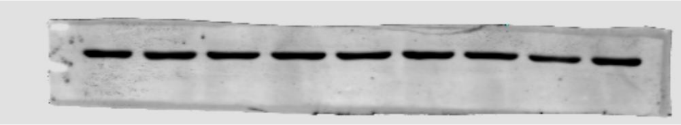

Fig. 5e LaminB

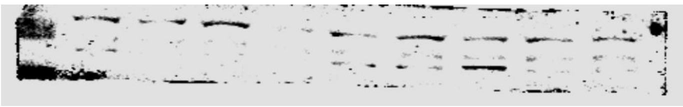

Fig. 5e NF-κB
